# HIV Infection and Persistence in Pulmonary Mucosal Double Negative T Cells *In Vivo*

**DOI:** 10.1128/JVI.01788-20

**Published:** 2020-11-23

**Authors:** Oussama Meziane, Syim Salahuddin, Tram N. Q. Pham, Omar Farnos, Amélie Pagliuzza, Ron Olivenstein, Elaine Thomson, Yulia Alexandrova, Marianna Orlova, Erwin Schurr, Petronela Ancuta, Élie Haddad, Nicolas Chomont, Eric A. Cohen, Mohammad-Ali Jenabian, Cecilia T. Costiniuk

**Affiliations:** a Infectious Diseases and Immunity in Global Health, Research Institute of the McGill University Health Centre, Montreal, Quebec, Canada; b Department of Biological Sciences, Université du Québec à Montréal, Montreal, Quebec, Canada; c Institut de Recherches Cliniques de Montréal, Montreal, Quebec, Canada; d Centre de Recherche du Centre Hospitalier de l’Université de Montréal, Montreal, Quebec, Canada; e Division of Respirology, Department of Medicine, McGill University, Montreal, Quebec, Canada; f Department of Microbiology and Immunology, McGill University, Montreal, Quebec, Canada; g Department of Human Genetics, McGill University, Montreal, Quebec, Canada; h Département de microbiologie, infectiologie et immunologie, Université de Montréal, Montreal, Quebec, Canada; i Research Center of CHU Sainte-Justine, Montreal, Quebec, Canada; j Department of Pediatrics, Université de Montréal, Montreal, Quebec, Canada; k Division of Infectious Diseases and Chronic Viral Illness Service, McGill University Health Centre, Montreal, Quebec, Canada; Emory University

**Keywords:** double negative (DN) T cells, HIV persistence, pulmonary mucosal immunity, lungs, T-cell immunity

## Abstract

The lungs are relatively unexplored anatomical human immunodeficiency virus (HIV) reservoirs in the antiretroviral therapy (ART) era. Double negative (DN) T cells are a subset of T cells that lack expression of CD4 and CD8 (CD4^−^ CD8^−^) and may have both regulatory and effector functions during HIV infection. Notably, circulating DN T cells were previously described as cellular HIV reservoirs. Here, we undertook a thorough analysis of pulmonary versus blood DN T cells of people living with HIV (PLWH) under ART. Bronchoalveolar lavage (BAL) fluid and matched peripheral blood were collected from 35 PLWH on ART and 16 uninfected volunteers without respiratory symptoms. Both PLWH and HIV-negative (HIV^−^) adults displayed higher frequencies of DN T cells in BAL versus blood, and these cells mostly exhibited an effector memory phenotype. In PLWH, pulmonary mucosal DN T cells expressed higher levels of HLA-DR and several cellular markers associated with HIV persistence (CCR6, CXCR3, and PD-1) than blood. We also observed that DN T cells were less senescent (CD28^−^ CD57^+^) and expressed less immunosuppressive ectonucleotidase (CD73/CD39), granzyme B, and perforin in the BAL fluid than in the blood of PLWH. Importantly, fluorescence-activated cell sorter (FACS)-sorted DN T cells from the BAL fluid of PLWH under suppressive ART harbored HIV DNA. Using the humanized bone marrow-liver-thymus (hu-BLT) mouse model of HIV infection, we observed higher infection frequencies of lung DN T cells than those of the blood and spleen in both early and late HIV infection. Overall, our findings show that HIV is seeded in pulmonary mucosal DN T cells early following infection and persists in these potential cellular HIV reservoirs even during long-term ART.

**IMPORTANCE** Reservoirs of HIV during ART are the primary reasons why HIV/AIDS remains an incurable disease. Indeed, HIV remains latent and unreachable by antiretrovirals in cellular and anatomical sanctuaries, preventing its eradication. The lungs have received very little attention compared to other anatomical reservoirs despite being immunological effector sites exhibiting characteristics ideal for HIV persistence. Furthermore, PLWH suffer from a high burden of pulmonary non-opportunistic infections, suggesting impaired pulmonary immunity despite ART. Meanwhile, various immune cell populations have been proposed to be cellular reservoirs in blood, including CD4^−^ CD8^−^ DN T cells, a subset that may originate from CD4 downregulation by HIV proteins. The present study aims to describe DN T cells in human and humanized mice lungs in relation to intrapulmonary HIV burden. The characterization of DN T cells as cellular HIV reservoirs and the lungs as an anatomical HIV reservoir will contribute to the development of targeted HIV eradication strategies.

## Abstract (precis)

Reservoirs of HIV during ART are the primary reasons why HIV/AIDS remains an incurable disease. Indeed, HIV remains latent and unreachable by antiretrovirals in cellular and anatomical sanctuaries, preventing its eradication. The lungs have received very little attention compared to other anatomical reservoirs despite being immunological effector sites exhibiting characteristics ideal for HIV persistence. Furthermore, PLWH suffer from a high burden of pulmonary non-opportunistic infections, suggesting impaired pulmonary immunity despite ART. Meanwhile, various immune cell populations have been proposed to be cellular reservoirs in blood, including CD4^−^ CD8^−^ DN T cells, a subset that may originate from CD4 downregulation by HIV proteins. The present study aims to describe DN T cells in human and humanized mice lungs in relation to intrapulmonary HIV burden. The characterization of DN T cells as cellular HIV reservoirs and the lungs as an anatomical HIV reservoir will contribute to the development of targeted HIV eradication strategies.

## INTRODUCTION

People living with human immunodeficiency virus (HIV) (PLWH) have a higher life expectancy today compared to that of the past 3 decades due to the widespread use of antiretroviral therapy (ART) (1). However, despite ART, normal immunity is not achieved in virally suppressed individuals, especially in the lungs, as demonstrated by a significantly higher prevalence of chronic lung diseases as well as viral and bacterial infections (2–5). Importantly, studies from the pre-ART years have shown that alveolar macrophages and pulmonary CD4 T cells harbor HIV (5). Following ART initiation, the virus remains detectable in the lungs (6). Our team recently reported that the frequency of infected CD4^+^ T cells within the lungs remains greater than in the blood of PLWH receiving long-term effective ART, suggesting that the lungs are implicated in the maintenance of long-lived HIV reservoirs (7).

While memory CD4^+^ T cells are known HIV reservoirs, other immune cells, including tissue macrophages and circulating double negative (DN) T cells, have also been shown to harbor latent HIV (8, 9). DN T cells are a subset of T cells devoid of CD4 and CD8 surface expression. DN T cells originate either from the thymus by escaping negative selection or are generated in the periphery through CD4 or CD8 downregulation in response to antigenic stimulation (10–20). Moreover, DN T cells are found in low frequencies in the peripheral blood, secondary lymphoid organs, and certain nonlymphoid tissues of healthy humans and rodents, while their proportions are increased in blood and tissues during autoimmune and inflammatory conditions (10, 12). Furthermore, both effector and immunoregulatory functions have been described for DN T cells (13, 21, 22). DN T cells are heterogeneous and may express T-cell receptor αβ (TCRαβ) or TCRγδ. Most (≈95%) human and mouse T cells are referred to as TCRαβ T cells due to their expression and rearrangement of α and β chains (10, 23). An uncommon subset of T cells (≈5%), which are mainly CD4^−^ CD8^−^, express the TCR γ and δ chains (10, 24). TCRαβ T cells are typically involved in adaptive immune responses, whereas TCRγδ T cells recognize antigens without presentation by major histocompatibility complex (MHC) molecules and respond directly to specific pathogens (10, 25).

During acute HIV infection, DN T cells play an immunoregulatory role by decreasing immune activation via the production of transforming growth factor β (TGF-β) and interleukin-10 (IL-10) (21, 22). In PLWH who progressed toward AIDS, the proportion of DN T cells in peripheral blood was double that of healthy controls (26). Meanwhile, DN T-cell frequency was decreased in patients with high viral load during early infection (27) and in PLWH who remain immunological nonresponders despite long-term ART (28). In another study, it was suggested that DN T cells may also contribute to Gag-specific immune response in HIV-exposed seronegative individuals with serodiscordant partners (29). Furthermore, in nonpathogenic simian immunodeficiency virus (SIV) infection of sooty mangabeys, DN T cells display T-helper functions and maintain their proliferative ability despite SIV infection (30). Therefore, DN T-cell frequencies change during the course of HIV infection and appear to play various roles in disease progression. One key area of research about DN T cells pertains to their contribution to viral persistence. HIV proteins Nef, Vpu, and Env are known to downregulate the expression of the CD4 receptor on the surface of infected CD4 T cells, which might in turn contribute to the generation of peripheral DN T cells (8, 18, 31–35). Importantly, HIV can be detected in DN T cells from peripheral blood and lymph nodes of PLWH even with undetectable plasma viral load (9, 36–38). Notably, HIV RNA has been detected in DN T cells, and infectious virus could be transmitted efficiently from DN T cells to uninfected cells (9). While DN T cells can express viral proteins from replication-defective proviruses, they may also produce replication-competent HIV, thus contributing to the persistence of a nonclassical cellular reservoir in ART-treated PLWH (8, 37).

Based on the role of DN T cells in HIV pathogenesis and their ability to harbor persistent HIV reservoirs, we aimed to assess the contribution of lung mucosal DN T cells to HIV reservoir persistence after long-term suppressive ART in PLWH without respiratory symptoms. In addition, to compensate for the impracticality of performing bronchoscopies during early HIV infection in PLWH, we assessed the dynamics of HIV infection and persistence in DN T cells in early versus late infection in the lungs of humanized bone marrow-liver-thymus (hu-BLT) mice.

(This work has been presented in part at the Conference on Retroviruses and Opportunistic Infections [CROI 2020], Boston, MA, USA, 2020.)

## RESULTS

### DN T cells with an effector memory phenotype are enriched in BAL fluid compared to those in blood in both ART-treated PLWH and uninfected individuals.

**FIG 1:**
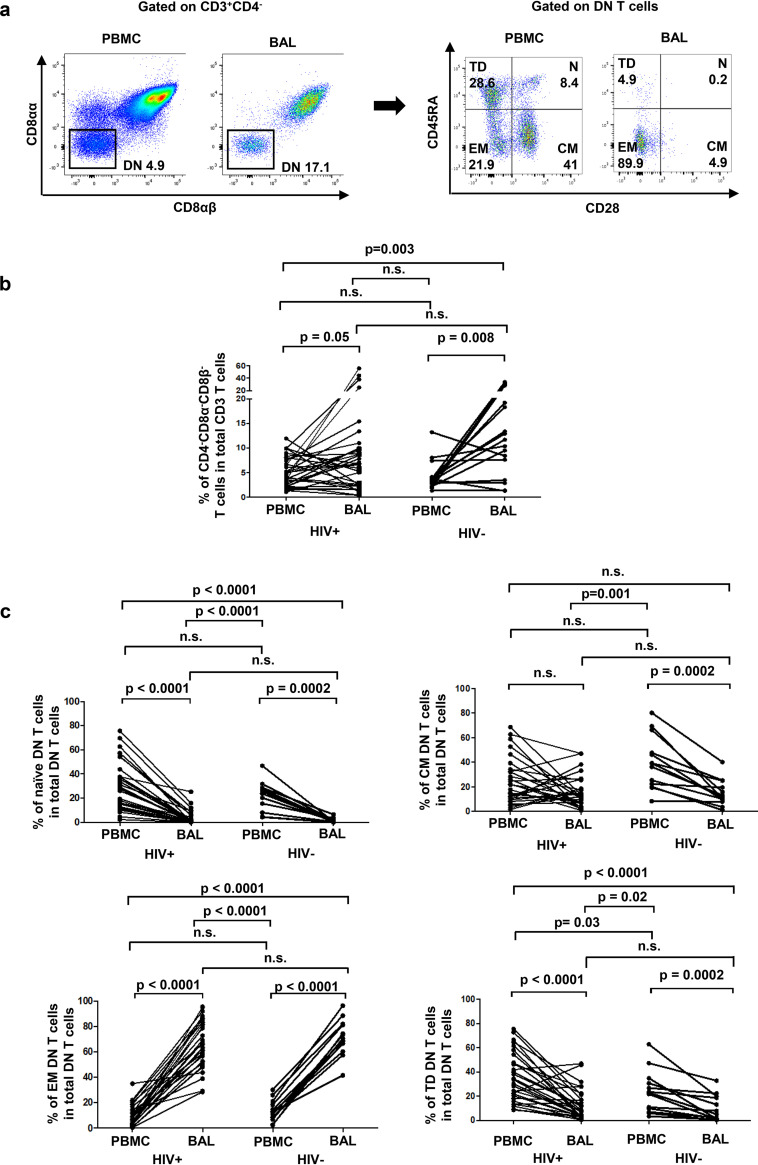
DN T cells are enriched in the BAL fluid compared to those in the blood and display mostly an effector memory phenotype.(a) Gating strategy used to define DN T cells as live CD3^+^ CD4^−^ CD8αα^−^ CD8αβ^−^ cells (left) and DN T-cell subsets as follows: naive (N, CD45RA^+^ CD28^+^), central memory (CM, CD45RA^−^ CD28^+^), effector memory (EM, CD45RA^−^ CD28^−^), and terminally differentiated (TD, CD45RA^+^ CD28^−^) (right). (b) Frequencies of DN T cells among total CD3 T cells in BAL fluid were compared to blood (HIV^+^, *n* = 32; HIV^−^, *n* = 16). (c) Frequencies of DN T-cell subsets among total DN T cells in BAL fluid were compared to those in blood (HIV^+^, *n* = 32; HIV^−^, *n* = 13). For the paired comparisons between BAL fluid and PBMCs within HIV^+^ or HIV^−^ groups, the Wilcoxon test was used, while for the comparisons of unpaired variables, the Mann-Whitney test was used.

We defined DN T cells as live CD3^+^ CD4^−^ CD8αα^−^ CD8αβ^−^ cells. The frequencies of DN T cells were higher in BAL samples than in blood in both groups of participants (HIV positive [HIV^+^], 10.3% ± 2.3% versus 5.2% ± 0.5%, respectively; HIV negative [HIV^−^], 13.3% ± 2.5% versus 4.3% ± 0.7%, respectively) (Fig. 1a and b). The proportions of terminally differentiated (TD) and naive (N) cells within total DN T cells were significantly lower in BAL fluid from both groups of participants than in matched blood (TD, HIV^+^, 12.4% ± 2.2% versus 35.3% ± 3.5% and HIV^−^, 9.7% ± 2.9% versus 23.6% ± 4.8%; N, HIV^+^, 3.4% ± 1% versus 29.5% ± 3.4% and HIV^−^, 1.6% ± 0.5% versus 22.3% ± 3.3%, respectively) (Fig. 1a and c). Conversely, a larger proportion of pulmonary mucosal DN T cells displayed an effector memory (EM) phenotype (HIV^+^, 65.6% ± 3.5% versus 10% ± 1.4%, respectively; HIV^−^, 71.5% ± 3.9% versus 13.6% ± 2.3%, respectively). The frequencies of central memory (CM) DN T cells from HIV^−^ participants, but not HIV^+^ participants, were lower in the BAL fluid than in blood (HIV^+^, 18% ± 2.6% versus 25% ± 3.2%, respectively; HIV^−^, 14.9% ± 2.9% versus 40% ± 6%; respectively) (Fig. 1a and c).

### Pulmonary mucosal DN T cells exhibit a unique phenotypic signature of activation, exhaustion/senescence, and trafficking/polarization markers in ART-treated PLWH.

**FIG 2:**
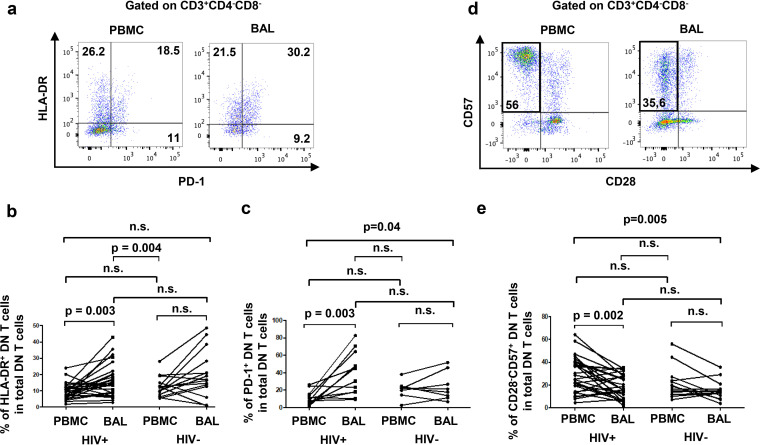
BAL fluid DN T cells express higher levels of activation marker HLA-DR and exhaustion molecule PD-1 and lower levels of senescence (CD57^+^ CD28^−^) in PLWH only. (a) Gating strategy to assess the expression of HLA-DR and PD-1 in DN T cells. (b) Frequencies of activated HLA-DR^+^ DN T cells among total DN T cells were compared in BAL fluid versus blood (HIV^+^, *n* = 26; HIV^−^, *n* = 15). (c) Frequencies of exhausted PD-1^+^ DN T cells among total DN T cells were compared in BAL fluid versus blood (HIV^+^, *n* = 12; HIV^−^, *n* = 8). (d) Gating strategy used to measure the frequencies of senescent DN T cells. (e) Frequencies of senescent DN T cells (CD28^−^ CD57^+^) were compared in BAL fluid versus blood (HIV^+^, *n* = 28; HIV^−^, *n* = 13). For the paired comparisons between BAL fluid and PBMCs within HIV^+^ or HIV^−^ groups, the Wilcoxon test was used, while for the comparisons of unpaired variables, the Mann-Whitney test was used.

**FIG 3:**
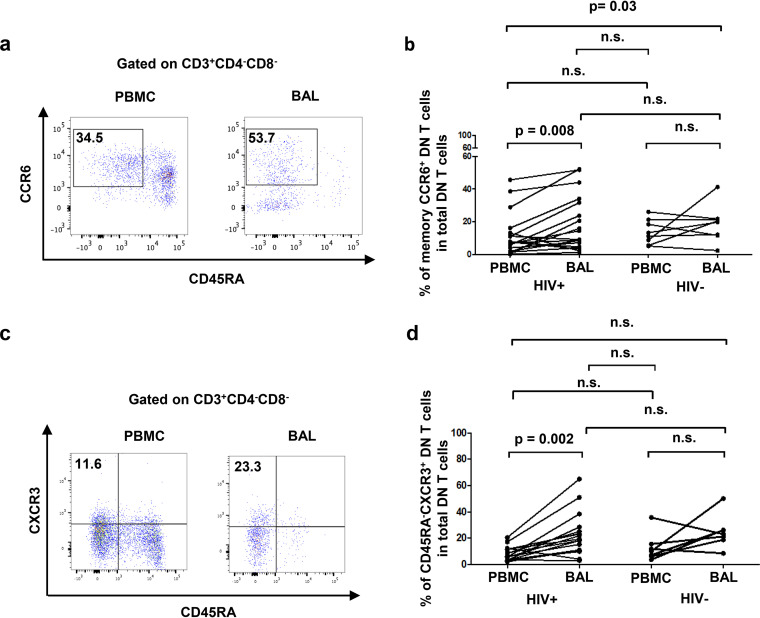
BAL fluid is enriched in memory CCR6^+^ and CXCR3^+^ DN T cells compared to matched blood. (a) Gating strategy used to assess the frequencies of memory CCR6^+^ DN T cells. (b) Frequencies of memory DN T cells expressing CCR6 among total DN T cells in BAL fluid versus blood (HIV^+^, *n* = 19; HIV^−^, *n* = 8). (c) Gating strategy used to assess the frequencies of memory CXCR3^+^ DN T cells. (d) Frequencies of memory DN T cells expressing CXCR3 among total DN T cells in BAL fluid versus blood (HIV^+^, *n* = 17; HIV^−^, *n* = 7). For the paired comparisons between BAL fluid and PBMCs within HIV^+^ or HIV^−^ groups, the Wilcoxon test was used, while for the comparisons of unpaired variables, the Mann-Whitney test was used.

PLWH had higher frequencies of HLA-DR^+^ activated DN T cells in BAL fluid than in blood (HIV^+^, 16.3% ± 2% versus 9.5% ± 1%, respectively; HIV^−^, 20.2% ± 3.7% versus 12.5% ± 1.6%, respectively) (Fig. 2a and b). Moreover, the frequencies of pulmonary DN T cells expressing PD-1, indicative of T-cell immune exhaustion and potential dysfunction, were enriched compared to blood in HIV^+^ participants (HIV^+^, 38.7% ± 7% versus 11.2% ± 2.2%, respectively; HIV^−^, 24.3% ± 5.8% versus 20.7% ± 3.5%, respectively) (Fig. 2a and c). Frequency of CD57^+^ CD28^−^ senescent DN T cells among total DN T cells were also assessed in both BAL fluid and blood, and lower frequencies of senescent DN T cells were observed in BAL fluid than in blood from PLWH (HIV^+^, 18.5% ± 2% versus 28.9 ± 2.9%, respectively; HIV^−^, 15.4% ± 2.4% versus 22% ± 3.9%, respectively) (Fig. 2d and e). Interestingly, we observed an enrichment in BAL fluid of a population of DN T cells with CCR6^+^ CD45RA^−^ (Fig. 3a and b) and CXCR3^+^ CD45RA^−^ (Fig. 3c and d) phenotypes in PLWH only (CCR6^+^ CD45RA^−^, HIV^+^, 18.3% ± 3.9% versus 11.7% ± 3% and HIV^−^, 18.8% ± 4% versus 13.7% ± 2.7%, respectively; CXCR3^+^ CD45RA^−^, HIV^+^, 22.7% ± 3.9% versus 8.2% ± 1.2% and HIV^−^, 24.9% ± 4.7% versus 13% ± 4%, respectively). Similar results have also been observed in pulmonary CD4^+^ T cells as described previously by our group (7). Since memory CCR6^+^ (39, 40), memory CXCR3^+^ (41, 42), and PD-1^+^ CD4 T cells (43) are known as preferential cellular reservoirs of HIV, these data suggest the potential of pulmonary mucosal DN T cells to serve as sanctuaries for HIV.

### Pulmonary mucosal DN T cells of ART-treated PLWH express relatively low levels of immunoregulatory markers.

**FIG 4:**
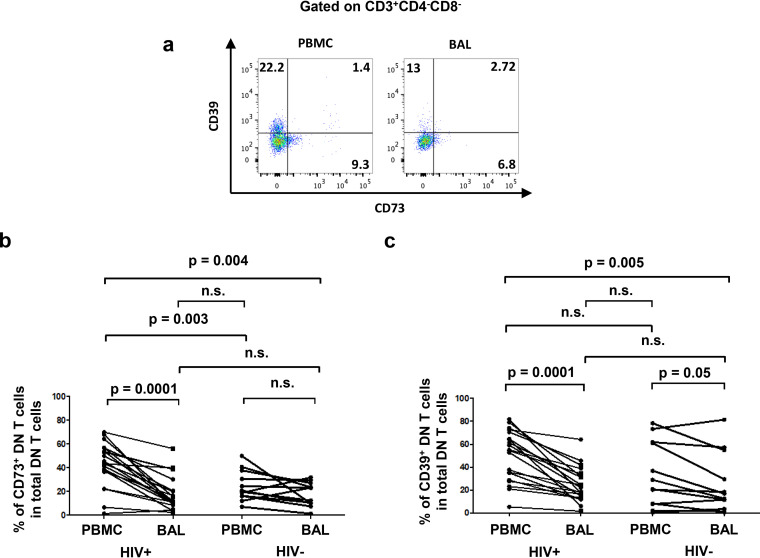
Lower frequencies of CD73^+^ and CD39^+^ DN T cells in BAL fluid versus blood. (a) Gating strategy used to assess the frequencies of DN T cells expressing CD73 or CD39. Frequencies of DN T cells expressing CD73 (b) and CD39 (c) in BAL fluid versus blood (HIV^+^, *n* = 20; HIV^−^, *n* = 12). For the paired comparisons between BAL fluid and PBMCs within HIV^+^ or HIV^−^ groups, the Wilcoxon test was used, while for the comparisons of unpaired variables, the Mann-Whitney test was used.

**FIG 5:**
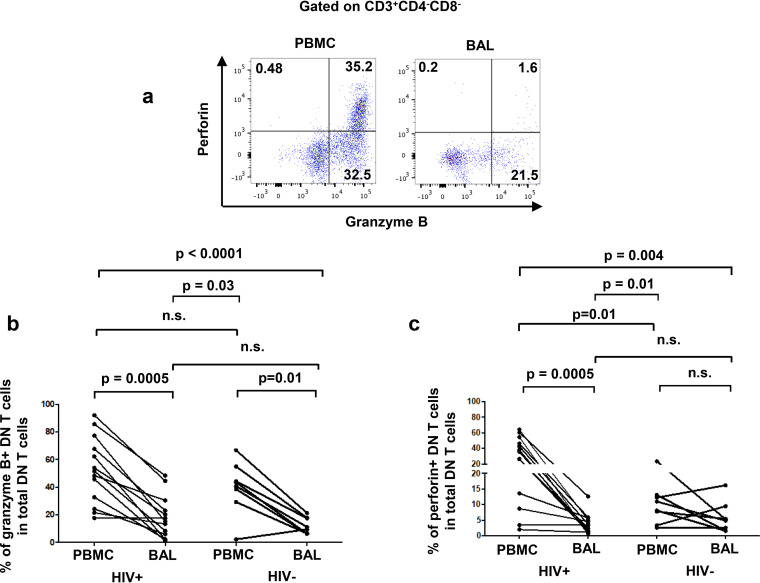
Fewer pulmonary DN T cells express granzyme B and perforin than their circulating counterparts. (a) Gating strategy used to analyze the frequencies of DN T cells expressing granzyme B and perforin. The frequencies of granzyme B^+^ DN T cells (b) and perforin^+^ DN T cells (c) among total DN T cells in BAL fluid were compared to those in blood (HIV^+^, *n* = 13; HIV^−^, *n* = 8). For the paired comparisons between BAL and PBMCs within HIV^+^ or HIV^−^ groups, the Wilcoxon test was used, while for the comparisons of unpaired variables, the Mann-Whitney test was used.

To assess the potential immunoregulatory functions of pulmonary mucosal DN T cells, we analyzed the expression of CD39 and CD73. These ectonucleotidases hydrolyze inflammatory ATP into immunosuppressive adenosine and are involved in inhibition of HIV-specific immune responses as we previously reported (44). We observed lower CD73^+^ and CD39^+^ DN T-cell frequencies in BAL fluid than in blood in PLWH (CD73^+^, HIV^+^, 18.4% ± 3.2% versus 43.3% ± 4.1 and HIV^−^, 17.5% ± 2.3% versus 24.5% ± 3.7%, respectively; CD39^+^, HIV^+^, 24.5% ± 3.4% versus 50.2% ± 5% and HIV^−^, 25.6% ± 7.4% versus 33.4% ± 8.2%, respectively) (Fig. 4a to c). We also assessed the expression of granzyme B and perforin since both molecules are needed for cytotoxic functions of DN T cells (45). We observed lower frequencies of DN T cells expressing granzyme B in BAL fluid than in blood in both PLWH and uninfected controls (HIV^+^, 18% ± 4.3% versus 52.4% ± 6.7%, respectively; HIV^−^, 12.1% ± 2.1% versus 40% ± 6.7%, respectively) (Fig. 5a and b). However, perforin-positive (perforin^+^) DN T cells were decreased in frequency in the BAL fluid compared to those in matched blood in PLWH only (HIV^+^, 3.7% ± 0.9% versus 32.5% ± 5.8%, respectively; HIV^−^, 5.9% ± 0.9% versus 10.2% ± 2.3%; respectively) (Fig. 5a and c).

### HIV persistence in pulmonary mucosal DN T cells in PLWH during viral-suppressive ART.

**FIG 6:**
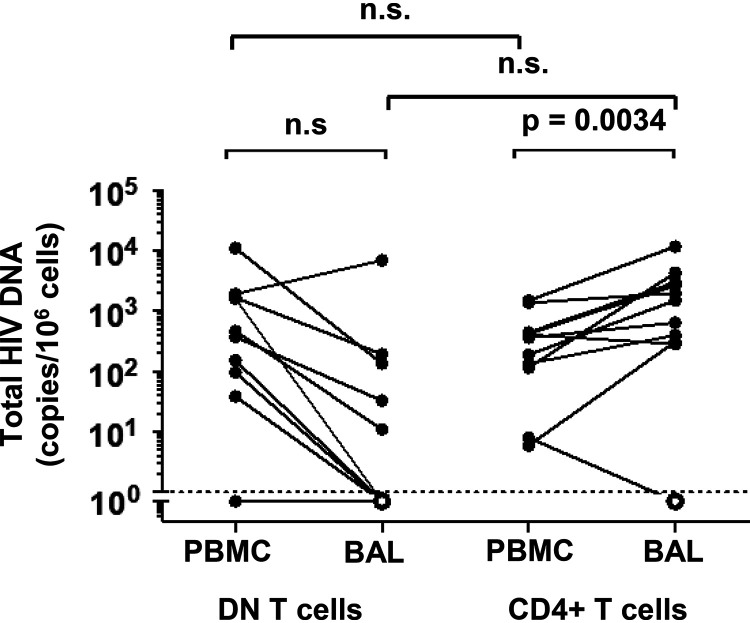
Presence of HIV DNA in DN T cells in PLWH under long-term ART. FACS-sorted DN and CD4^+^ T cells from BAL fluid and matched PBMCs of *n* = 16 ART-suppressed were assessed. Only frequencies of cells harboring HIV DNA in DN and CD4 T-cell specimens for which we obtained at least 3,000 CD3 copies are shown. Samples with undetectable HIV DNA are plotted as open symbols.

We recently reported that pulmonary mucosal CD4^+^ T cells found in BAL fluid harbor significantly higher HIV DNA than their circulating counterparts (7). To investigate the role of DN T cells from the lung as a viral reservoir, we measured HIV DNA in fluorescence-activated cell sorter (FACS)-sorted live CD3^+^ CD4^−^ CD8^−^ DN T cells and matched CD4^+^ T cells from BAL fluid and blood of 16 individual PLWH on ART. As expected, HIV DNA was detected in blood and pulmonary CD4^+^ T cells from the majority of the 16 participants (Fig. 6). We obtained a sufficient number of sorted BAL fluid DN T cells for HIV DNA quantification from 7 of the 16 participants. Among these, 5 had measurable levels of HIV DNA, indicating that lung DN T cells harbor HIV DNA in the majority of virally suppressed individuals (Fig. 6).

### Early and preferential establishment of HIV infection in DN T cells in the lungs of hu-BLT mice.

**FIG 7:**
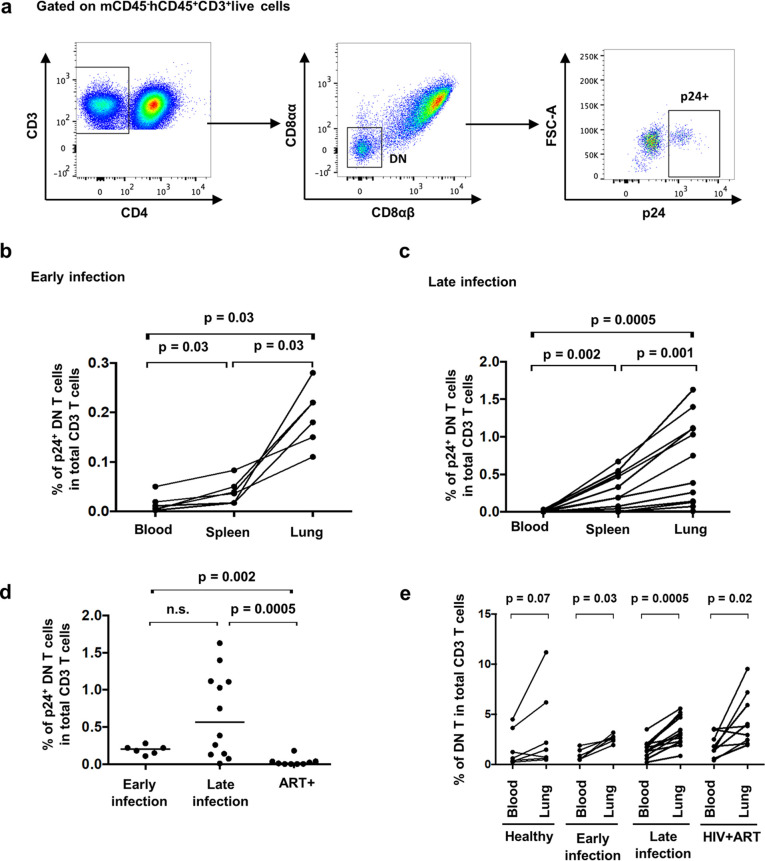
Higher frequencies of infected p24^+^ DN T cells in lungs than blood and spleen in both early and late HIV infection of humanized BLT mice. (a) Gating strategy used to assess the frequencies of total DN T cell and infected DN T cells in hu-BLT mice. Frequencies of p24^+^ DN T cells in the lungs were compared to those in the blood and spleen of hu-BLT mice in early (b) and late (c) stages of HIV infection. (d) Frequencies of infected p24^+^ DN T cells were compared in the lungs of hu-BLT mice during the early and late phases of HIV infection as well as under ART treatment. (e) Frequencies of DN T cells in the lungs of hu-BLT mice compared to those in the blood of healthy and HIV-infected mice in early and late HIV infection and following ART (healthy, *n* = 7; early HIV infection, *n* = 6; late HIV infection, *n* = 12; ART-treated, *n* = 9).

Although HIV reservoir persistence in DN T cells is well established in ART-treated PLWH, the timing of their infection and the establishment of HIV reservoirs within lung DN T cells remain unexplored. Using a hu-BLT mouse model of HIV infection (46), we investigated the establishment of HIV infection of DN T cells in the lungs compared to that in the blood and spleen during early and late phases. Three weeks postinfection of hu-BLT mice by HIV NL4.3-ADA-GFP is defined as the early phase of infection and 7 to 12 weeks postinfection is defined as the later phase of infection in this mouse model as previously reported (47, 48). Six hu-BLT mice were sacrificed during the early phase with 26,000 to 60,000 HIV copies/ml of plasma, and 12 animals were sacrificed in the later phase when the infection had plateaued (median, 10^6^ copies/ml of plasma). We found an enrichment of infected DN T cells in the lungs compared to that in the spleen and the blood during the early phase and maintained during the later phase (Fig. 7a to c). HIV-p24^+^ DN T cells were absent in the lungs of ART-treated mice (Fig. 7d). Nevertheless, similar to data obtained with cells from ART-treated PLWH, the frequency of DN T cells in the lungs remained higher compared to that in the blood in all hu-BLT study groups regardless of HIV status and treatment (Fig. 7e). Overall, our results demonstrate that the enrichment of HIV-infected DN T cells is consistently present in the lungs of hu-BLT mice during both early and late infection. Similar to humans, the frequencies of total DN T cells remain higher in the lungs than in blood regardless of HIV infection.

## DISCUSSION

Higher burden of pulmonary inflammatory illnesses and lung infections in PLWH, despite ART, highlights the need for a better understanding of regulation of pulmonary mucosal immunity. In addition, the persistence of cellular and anatomical reservoirs of HIV is the main reason why HIV infection remains incurable despite the success of ART. Due to difficulties in specimen accessibility, the lungs compared to other anatomical reservoirs have been minimally investigated in the ART era. Nevertheless, the lungs represent important immunological effector sites with characteristics ideal for HIV persistence (5). We recently reported a greater HIV reservoir size in CD4^+^ T cells from the lung mucosa than the blood of PLWH on long-term ART (7). In the present study performed on BAL fluid and matched peripheral blood from PLWH on long-term ART without respiratory symptoms and from uninfected controls, we found that DN T cells were significantly enriched in the lung mucosa compared to those in blood regardless of HIV status. This could be explained, in part, by the fact that the lungs serve as a preferential compartment for DN γδ T-cell homing during perinatal development (49). Such an accumulation of DN T cells in the lung mucosa is of importance, as these cells are heterogeneous by origin and could display various immunological functions during HIV/SIV infections, including both T-helper or immunoregulatory activity (13, 21, 22). Furthermore, human DN T cells also decrease tissue homing capacity and modulate effector functions of CD4^+^ T cells (50). In contrast, CD8-derived human TCRαβ DN T cells display a proinflammatory effector phenotype (20).

We found that, compared to those in blood, pulmonary mucosal DN T cells from both ART-treated PLWH and uninfected individuals displayed mostly an EM phenotype. This observation is expected since EM T cells migrate into nonlymphoid tissues as long-lived memory cells in response to infection or inflammation (51, 52), and the lungs are immunological effector tissues which are continuously exposed to various antigens. Interestingly, EM T cells have been described as the main subset harboring HIV DNA and HIV RNA in the gut mucosal tissues of ART-treated PLWH (40, 53). Furthermore, in blood of PLWH on ART, EM T cells encompass the majority of intact and replication-competent HIV DNA among other memory T cells (54–56). The dominance of EM T cells supports the hypothesis that DN T cells in the lung mucosa of HIV-infected individuals are not recent migrants from the thymus and may instead have originated extrathymically as a result of HIV infection since Nef and Vpu are known to downregulate CD4 (8, 18, 31–33). Alternatively, in some autoimmune diseases, human TCRαβ DN T cells have been shown to clonally originate from CD8^+^ T cells (11). To clarify the identity of mucosal DN T cells, further investigations, such as TCR repertoire sequence diversity or single-cell transcriptomic analysis, are needed to determine if DN T cells in the lungs originated from CD4 or CD8 T cells.

In ART-treated PLWH compared to uninfected individuals, we observed higher frequencies of HLA-DR^+^ and PD-1^+^ DN T cells suggesting their greater levels of immune activation and exhaustion, respectively. Cellular immune activation may contribute to HIV persistence through promoting HIV replication and enhancing susceptibility of bystander cells to infection (57), in addition to being implicated in driving a proinflammatory environment within the lungs, contributing to chronic lung disease in PLWH (1, 5). Meanwhile, it is well documented that PD-1 contributes to the establishment and persistence of HIV-1 latency (58) and PD-1 blockade potentiates HIV latency reversal (43). PD-1 and Helios expression can further distinguish TCRαβ^+^ DN T cells derived from self-reactive CD8 T cells (59). In PLWH, pulmonary mucosal DN T cells displayed a lower CD57^+^ CD28^−^ senescent phenotype than those in the blood. This is in line with the dominant EM phenotype observed among BAL fluid DN T cells at the expense of their end-stage TD subset. Highly differentiated and senescent CD57^+^ CD28^−^ T cells are proinflammatory (60), and CD57 expression defines replicative senescence and antigen-induced apoptotic death of CD8 T cells during HIV infection (61). Accordingly, and of relevance to our findings, it has been shown that DN T cells maintain their proliferative capacity and effector T-helper function in SIV infection of sooty mangabeys (30).

We also assessed the frequencies of CCR6-expressing DN T cells in the lungs compared to those in peripheral blood. Within the CD4^+^ T-cell compartment, memory CCR6^+^ Th17 cells were found to be preferentially infected very early following infection and harbor high levels of replication-competent HIV DNA compared to CCR6^−^ T cells (40, 62–64). In addition, DN T cells in the lungs of mice were shown to express CCR6 and to produce interleukin-17 (IL-17) (49). In line with these findings and our previous report on the enrichment of memory CCR6^+^ CD4^+^ T cells within the lungs, we found that memory CCR6^+^ DN T cells were enriched in the BAL fluid of PLWH compared to those in blood (7). These CCR6-expressing cells within the memory DN T-cell compartment may, therefore, play a substantial role in maintaining the HIV burden within the lungs, akin to their CD4^+^ T-cell counterparts. Besides CCR6, another chemokine receptor, CXCR3, has recently been described as an additional marker of HIV reservoir in PLWH under effective ART (41). Similar to CCR6 expression, memory CXCR3^+^ DN T cells were enriched within the lung mucosa versus those in blood. CXCR3 has also been identified as a marker of T-cell homing into the lung tissue (65). Thus, besides contributing to HIV persistence, higher CXCR3 expression by pulmonary DN T cells suggests that the accumulation of DN T cells might be explained by the recruitment of T cells into the lung mucosa.

DN T cells play an important immunoregulatory role by decreasing immune activation during acute HIV infection via TGFβ and IL-10 production (21, 22), and lower frequency of DN T cells is associated with higher viral load during acute infection (27, 28). We therefore evaluated the expression of CD39 and CD73, two ectonucleotidases expressed by regulatory T cells, which together convert inflammatory ATP into anti-inflammatory adenosine. We previously showed that the adenosine pathway is involved in the inhibition of anti-HIV-specific effector T-cell responses (44). In addition, it has been demonstrated that mouse DN T cells in the lungs express high levels of CD39, enhancing the production of the suppressor cytokine IL-10 (49, 66). However, in our study, human pulmonary mucosal DN T cells express lower levels of these ectonucleotidases than those in the blood. Moreover, cytotoxic activity has been described as an immunoregulatory function of DN T cells (45, 67). Interestingly, important decreases in the expression of perforin and granzyme B were observed in BAL fluid DN T cells versus that in blood. Similar to our results, an important decrease in perforin and granzyme B expression by gut mucosal CD8 T cells and their cytotoxicity has been previously reported regardless of HIV infection (68, 69). Therefore, lower cytotoxic capacity of tissue-resident T cells might be an adaptation to their microenvironment to preserve mucosal barrier integrity (70). Overall, although it remains to be confirmed, our observations suggest that pulmonary DN T cells exhibit lower immunoregulatory capabilities than their peripheral counterparts, especially in PLWH.

To determine whether the high expression of cellular markers of HIV reservoirs by pulmonary mucosal DN T cells coincides with HIV persistence in these cells, we measured HIV DNA levels in all specimens for which we had sufficient numbers of FACS-sorted DN T cells; no significant differences were observed in HIV DNA levels between DN and CD4^+^ T cells in BAL fluid. Pertinently, in the female genital mucosa, a subset of DN T cells expressing the lymphocyte-activating gene 3 (LAG-3) was reported to be greatly permissive to HIV (71). In addition, blood DN T cells of PLWH have been identified as persistent HIV reservoirs that carry HIV Gag protein despite ART and contribute to viral persistence (8). Furthermore, Nef, a viral protein known to efficiently downregulate CD4 expression, persists in the lungs of aviremic PLWH, causing pulmonary vascular pathologies via the induction of endothelial cell apoptosis (72). In order to better understand the dynamics of HIV-infected pulmonary DN T cells during different phases of infection, we used a hu-BLT mice model (46), as it is not feasible to perform bronchoscopies during acute infection in PLWH. Interestingly, when compared to blood and spleen as a lymphoid tissue, the lungs were enriched in p24^+^ DN T cells in both early and late HIV infection stages, suggesting that the lungs are favorable tissues for HIV seeding within DN T cells during acute infection. Indeed, HIV has been isolated from the BAL fluid of untreated PLWH during early infection (5). Although ART initiation in hu-BLT mice suppressed viral replication in pulmonary DN T cells, the frequencies of DN T cells within the lungs remained consistently higher than in blood.

This study has some limitations which merit mentioning. Firstly, within BAL fluid, alveolar macrophages are the dominant population, while the number of lymphocytes is relatively low. Notably, low frequencies of DN T cells restricted the number of assessments we could perform in a given BAL specimen. Thus, we prioritized phenotypic analysis and HIV DNA quantification. In this context, the quantification of intact HIV DNA by DNA sequencing and of replication-competent reservoirs by viral outgrowth assays remains to be performed in upcoming studies. Moreover, considering the unique immunological features of DN T cells revealed in this study, it will be important to carry out future systems biology studies at single cell level for an in-depth characterization of these cells during HIV infection. Finally, although we did not perform genetic sequencing of HIV variants in BAL fluid versus blood, we anticipate the existence of phylogenetic differences in HIV sequences from these two different anatomical compartments.

Taken together, considering the dual immunological effector/regulatory roles of DN T cells, our study provides evidence suggesting peculiar dynamics and phenotypes of DN T cells in pulmonary mucosal tissues of PLWH. Our results demonstrate an enrichment of DN T cells within the pulmonary mucosal tissue in both ART-treated PLWH and uninfected individuals. In addition, our findings provide additional support for the lungs as anatomical HIV sanctuary tissues in PLWH despite long-term viral-suppressing ART. We also showed that HIV is seeded in pulmonary DN T cells early following infection in hu-BLT mice and that HIV reservoirs persist in pulmonary mucosal DN T cells expressing a unique phenotype in ART-treated PLWH. These findings are of fundamental relevance for understanding the role of DN T cells in pulmonary mucosal immunity and viral persistence.

## MATERIALS AND METHODS

### Study population.

Thirty-five PLWH under ART treatment with undetectable plasma HIV viral load for at least 3 years and 16 HIV-uninfected participants were enrolled at McGill University Health Centre (Montreal, Canada). All participants recruited did not exhibit any respiratory symptoms or active infection. Exclusion criteria included asthma, chronic obstructive pulmonary disease, or any acute respiratory symptoms. Clinical characteristics of study participants are described in Table 1.

**TABLE 1:** Participant characteristics at time of bronchoscopy

Factor^*a*^	Study population (*n* = 51)	
	HIV^+^ (*n* = 35)	HIV^−^ (*n* = 16)
Demographic factors		
Median age (yrs [IQR])	54 (50.0–59.0)	52 (29.75–60.75)
Male sex (no. [%])	29 (82.9)	16 (100)
Ethnicity (no. [%])		
Caucasian	27 (77.1)	15 (93.8)
Black/Caribbean	2 (5.7)	0 (0)
Black/African	3 (8.6)	0 (0)
Black/Haitian	1 (2.9)	0 (0)
Hispanic	2 (5.7)	0 (0)
South East Asian	0 (0)	1 (6.3)
HIV and immune-related factors		
Median duration of HIV infection (yrs [IQR])	16 (12.0–25.0)	
Median duration of time since viral load suppressed (yrs [IQR])	9 (4.0–12.0)	
Antiretroviral regimen components (no. [%])		
Integrase inhibitor	21 (60)	
NRTI	33 (94.3)	
NNRTI	7 (20)	
PI	10 (28.6)	
Median CD4 count (cells/mm^3^ [IQR])	537.0 (412.0–808.0)	607.5 (336.5–796.8)
CD4/CD8 ratio (IQR)	0.7 (0.5–0.95)	2.1 (1.3–3.2)
Median CD8 count (cells/mm^3^ [IQR])	779.0 (535.5–1095.0)	272.5 (144.3–501.3)
Pulmonary coinfection/opportunistic infection history (no. [%])		
Previous pneumocystis pneumonia	2 (5.7)	
Previous Mycobacterium avium pneumonia	1 (2.9)	
Kaposi’s sarcoma	1 (2.9)	
Latent tuberculosis infection	1 (2.9)	
Lifestyle factors		
Tobacco smoker (no. [%])		
Yes	13 (37.1)	5 (31.3)
No	22 (62.9)	11 (68.8)
Cannabis smoker (no. [%])		
Current	6 (17.1)	1 (6.3)

a IQR, interquartile range; NRTI, nucleoside reverse transcriptase inhibitor; NNRTI, nonnucleoside reverse transcriptase inhibitor; PI, protease inhibitor.

### Ethical consideration.

This study was ethically approved by the Research Institute of the McGill University Health Centre (no. 15-031), Université du Québec à Montréal (no. 602), and CHUM-Research Centre (no. 15-180). All participants signed a written informed consent.

### Bronchoalveolar lavage fluid and blood collection.

Bronchoscopies were performed to obtain up to 100 ml of BAL fluid. BAL specimen cells and matched peripheral blood mononuclear cells (PBMCs) were then isolated as we previously reported (7, 73). Of note, due to the limited numbers of purified BAL specimen cells, interindividual variations, and low DN T-cell frequencies, we were not able to perform all study measures as described below in all individuals, and we prioritized the measures to be assessed based on the available cell number for each study individual.

### Flow cytometry phenotyping.

Half a million BAL cells or PBMCs were stained with a cocktail of antibodies for DN T-cell phenotyping. To eliminate dead cells from the analysis, we stained cells with Aqua viability stain (Invitrogen) and define DN T cells as Aqua-CD3^+^ CD4^−^ CD8αα^−^ CD8αβ^−^ cells. Anti-HLA-DR was used as a marker of activation. Anti-CD28, anti-CD45RA, and anti-CD57 were used to identify naive, CM, EM, TD, and senescent DN T cells. Anti-CCR6 and anti-CXCR3 were included to identify previously described cellular HIV reservoirs, as well as T-cell homing in lung tissue using the latter (65). Intracellular stainings were performed using anti-perforin and anti-granzyme B to assess cytotoxicity phenotype of DN T cells. References for all antibodies used for this study are described in Table 2.

**TABLE 2:** List of antibodies used for flow cytometry

Antibody	Fluorochrome	Clone	Reference	Catalog no.
CCR6	PE	11A9	BD Pharmingen	551773
CD28	PE-Cy5	CD28.2	BD Pharmingen	560684
CD3	Alexa F 700	UCHT1	BD Pharmingen	557943
CD39	BV-711	TU66	BD Horizon	563680
CD4	BV 605	RPA-T4	BD Horizon	562659
CD4	APC	RPA-T4	BD Pharmingen	555349
CD45RA	BV786	HI100	BD Horizon	563870
CD57	BV605	NK-1	Biolegend	393304
CD73	FITC	AD2	BD Pharmingen	561254
CD8	BV605	SK1	BD Horizon	564116
CD8αα	APC/H7	SK1	BD Pharmingen	560179
CD8αβ	PE-cy7	SIDI8BEE	eBioscience	25-5273-42
CXCR3	PE-Cy5	L201C6/CXCR30	BD Horizon	563156
hCD45	APC/Cy7	HI30	Biolegend	304014
hCD45	PE/Cy7	HI30	BD Bioscience	557748
HLA-DR	BV421	G46-6	BD Horizon	562804
Live/Dead	Aqua vivid		Thermo Fisher	L34957
mCD45	PE-Dazzle	30-F11	BioLegend	103146
p24	PE	KC57	Beckman Coulter	6604667
PD1	PE	EH12.2H7	BD Pharmingen	557946

### Fluorescence-activated cell sorting of DN T cells.

A fraction of cells from BAL fluid and matched blood was used to isolate CD4^+^ and DN T cells by FACS. Both BAL specimen cells and PBMCs were stained with Live/Dead blue dye, -PE-Cy7 anti-CD45, Alexa 700 anti-CD3, APC anti-CD4, and BV605 anti-CD8. CD4^+^ and DN T cells were FACS-sorted using a BD FACSAria as we previously described (73).

### HIV DNA quantification.

FACS-sorted DN T cells and CD4^+^ T cells were lysed using QIAamp DNA minikit according to the manufacturer’s instructions (Qiagen). HIV DNA quantification was performed in triplicate using an ultrasensitive reverse transcriptase PCR (RT-PCR)-adapted protocol as we previously described (7, 73, 74). Only samples for which at least 3,000 cells were available were included in the analysis.

### Infection and analysis of humanized mice.

Humanized bone marrow-liver-thymus (hu-BLT) mice were generated and infected with HIV NL4.3-ADA-GFP as we previously described (46). To characterize DN T cells in early and late infections, mice were sacrificed at 3 and 7 to 12 weeks postinfection, respectively. To assess whether DN T cells can support viral persistence during ART, a group of mice was treated with ART (raltegravir, 70 mg/ml; emtricitabine, 166 mg/ml; and tenofovir, 170 mg/ml) or phosphate-buffered saline (PBS) as control for 3 to 6 weeks (47). In all cases, spleen and lung tissues were harvested; cells from blood and the tissues were isolated as previously described (46). At sacrifice, all ART-treated mice were virally suppressed (<40 HIV RNA copies/ml of plasma).

### Statistical analyses.

GraphPad prism v6.01 (CA, USA) was used to perform statistical analyses. The Wilcoxon matched-pair signed-rank test and Mann-Whitney test were used to compare paired and unpaired variables, respectively. The *P* value is presented in figures, and n.s. denotes a statistically insignificant comparison. In the text, reported results follow the mean ± standard error of the mean (SEM) format.
